# GWAS reveals novel loci and identifies a *pentatricopeptide repeat-containing protein (CsPPR)* that improves low temperature germination in cucumber

**DOI:** 10.3389/fpls.2023.1116214

**Published:** 2023-04-26

**Authors:** Caixia Li, Shaoyun Dong, Diane M. Beckles, Xiaoping Liu, Jiantao Guan, Xingfang Gu, Han Miao, Shengping Zhang

**Affiliations:** ^1^ State Key Laboratory of Vegetable Biobreeding, Institute of Vegetables and Flowers, Chinese Academy of Agricultural Sciences, Beijing, China; ^2^ Department of Plant Sciences, University of California Davis, Davis, CA, United States

**Keywords:** cucumber, low temperature germination, GWAS, polygenic, *CsPPR*

## Abstract

Low temperatures (LTs) negatively affect the percentage and rate of cucumber (*Cucumis sativus* L.) seed germination, which has deleterious effects on yield. Here, a genome-wide association study (GWAS) was used to identify the genetic loci underlying low temperature germination (LTG) in 151 cucumber accessions that represented seven diverse ecotypes. Over two years, phenotypic data for LTG i.e., relative germination rate (RGR), relative germination energy (RGE), relative germination index (RGI) and relative radical length (RRL), were collected in two environments, and 17 of the 151 accessions were found to be highly cold tolerant using cluster analysis. A total of 1,522,847 significantly associated single-nucleotide polymorphism (SNP) were identified, and seven loci associated with LTG, on four chromosomes, were detected: *gLTG1.1*, *gLTG1.2*, *gLTG1.3*, *gLTG4.1*, *gLTG5.1*, *gLTG5.2*, and *gLTG6.1* after resequencing of the accessions. Of the seven loci, three, i.e., *gLTG1.2*, *gLTG4.1*, and *gLTG5.2*, showed strong signals that were consistent over two years using the four germination indices, and are thus strong and stable for LTG. Eight candidate genes associated with abiotic stress were identified, and three of them were potentially causal to LTG: *CsaV3_1G044080* (a pentatricopeptide repeat-containing protein) for *gLTG1.2*, *CsaV3_4G013480* (a RING-type E3 ubiquitin transferase) *for gLTG4.1*, and *CsaV3_5G029350* (a serine/threonine-protein kinase) for *gLTG5.2.* The function for *CsPPR* (*CsaV3_1G044080*) in regulating LTG was confirmed, as *Arabidopsis* lines ectopically expressing *CsPPR* showed higher germination and survival rates at 4°C compared to the wild-type, which preliminarily illustrates that *CsPPR* positively regulates cucumber cold tolerance at the germination stage. This study will provide insights into cucumber LT-tolerance mechanisms and further promote cucumber breeding development.

## Introduction

Cucumber (*Cucumis sativus* L.), as one of the most economically important vegetable crops, is widely cultivated on ~2.3 Mha globally and its production reached 91.26 M metric tons in 2020 (FAO, 2020). Cucumber, however, is a temperature-sensitive crop that is easily injured by low temperatures (LTs) (Cabrera et al., 1992). LT is one of the most common abiotic stresses; it can affect cucumber seed architecture and germination, slow the growth rate, delay seed maturation, decrease the rate of seed set, and even indirectly affect reproduction, which collectively threatens cucumber production and quality (Nienhuis et al., 1983; Røeggen, 1987). When cucumber seeds encounter LTs, either in the soil or in irrigation water, the percentage of seeds that germinate decreases, and more seriously, LTs can cause seed death (Smeets and Wehner, 1997). Clarifying the genetic basis of cucumber LT germinability, will enable molecular breeding vital to improving yield in cold regions, as well as extending the cultivated area from low- to high-altitudes.

Previous studies reported that low temperature germination (LTG) is a complex quantitative trait loci (QTL) commonly controlled by multiple genes (Kłosińska et al., 2013). Germination rate (GR), germination energy (GE), germination index (GI), and radicle length (RL) were the common used LTG evaluation indices (Song et al., 2018; Yagcioglu et al., 2019). To date, only three QTL analyses were performed in cucumber using these indices. First, Dong (2017) identified eight QTLs using F_2:3_ families. Next, three QTLs (*qLTG1.1*, *qLTG2.1* and *qLTG4.1*) were detected using Recombinant Inbred Lines (RILs) in 2018 (Song et al., 2018; Li et al., 2022a). Subsequently, Yagcioglu et al. (2019) also identified three QTLs (*qLTG1.2*, *qLTG2.1* and *qLTG4.1*) using RILs and F_2:3_ families. Collectively these studies provided important information on LTG-related genetics and accelerated the development of cucumber breeding programs.

A genome-wide association study (GWAS) is an effective way to identify complex quantitative traits, and it has been widely used in crops. In rice, Sales et al. (2017) showed that 31 markers were related to growth rate and germination rate at 25°C and 15°C using 200 diverse germplasms and 1672 single nucleotide polymorphisms (SNPs). Schläppi et al. (2017) evaluated 202 core germplasms and identified two new loci, i.e., *qLTSS3-4* and *qLTSS4-1*, using seedling survival rate as an evaluation index under LT. A member of the 14-3-3 gene family (*GF14h*), was associated with changes in the germination rate of rice under optimal temperature conditions using GWAS (Yoshida et al., 2022). In sorghum, based on five evaluation indicators (stem weight, root weight, stem length, root length and anthocyanin content) at LT, transcription factors *Sb06g024820* and *Sb05g001215* were identified as candidate genes capable of regulating LT responses (Chopra et al., 2017). In maize, *Zm00001d017932*, a MADS-transcription factor 26 (*MADS26*), were verified as affecting seed germination *via* an association GWAS analysis of 300 lines and 24 transcriptome libraries analysis (Ma et al., 2022). In cotton, using GWAS mapping with 200 accessions, *Gh_A01G1740* (*GhSAD*), which encodes a short-chain alcohol dehydrogenase, was found to confer cold tolerance (Ge et al., 2022). Further, *Gh_D09G0189* (*GhSAL1*), a bifunctional protein, was identified as affecting cold tolerance in upland cotton at the seedling emergence stage, using 200 accessions from five ecological distributions (Shen et al., 2023). With the development of cucumber genome sequencing, GWAS has also been widely used to accelerate genes related to LTG. GWAS has also been successfully used to detect many QTLs in cucumber, for example, for biotic and abiotic stress resistance, and for fruit quality traits. For biotic stress resistance, novel powdery mildew (PM) resistance genes, i.e., *Csa5G453160* and *Csa5G471070*, found on chromosome 5 were detected using a set of 264 from a core germplasm collection (Lee et al., 2020). Meanwhile, eight PM-resistance, five downy mildew resistance and four gummy stem blight resistance genes were putatively identified (Liu et al., 2020b; Liu et al., 2021; Han et al., 2022). For abiotic stress, Zhang et al. (2019) evaluated heat tolerance with a collection of 96 accessions and found two loci - *GGI4.1* and *GGI5.1* using GR, GE and GI. Wei et al. (2019) detected seven GWAS locus (*gHII4.1*, *gHII4.2*, *gHII5.1*, *gHII5.2, gHII6.1*, *gHII6.2* and *gHII7.1*) using a heat injury index. Wang et al. (2019) detected five LT-tolerance loci (*gLTS1.1, gLTS3.1*, *gLTS4.1* and *gLTS5.1*) on Chr.1, Chr.3, Chr.4 and Chr.5, respectively using low temperature injury indices. Seven loci (*gST2.1*, *gST3.1*, *gST4.1*, *gST4.2, gST5.1, gST6.1)* associated with salt tolerance in cucumber seedlings were repeatedly detected (Liu et al., 2022a). For quality traits, the ultra-high fruit spine density formation genes (*fsd6.2*) (Bo et al., 2019a) and the green flesh formation gene (*qgf5.1* and *qgf3.1*) (Bo et al., 2019b) were identified using a group of 115 cucumber accessions from a core germplasm collection. However, there have been no reported studies investigating LTG tolerance using the GWAS approach.

In this study, we uncovered the genetic architecture of LTG in re-sequenced lines of a 151 core cucumber germplasm population which represented genotypes of diverse origins and ecotypes. Low temperature tolerance at the cucumber germination stage was assessed *via* four indices and 1,522,847 SNP markers. Three loci related to LTG were detected in two different environments by GWAS. Moreover, three novel candidate genes were discovered and assessed via functional annotation, sequence alignment and expression analysis. Finally, the *CsaV3_1G044080* gene within the *gLTG1.2* locus, encoded a pentatricopeptide repeat-containing protein (*CsPPR*) was further verified; When ectopically expressed in *Arabidopsis*, it engendered better LTG in the transgenic lines. The objectives of this study are to (i) enrich the diverse genetic variation and screen low temperature tolerance accession; (ii) provide insights into the genetic basis of cucumber LTG tolerance, as well as resources for the genetic improvement of cucumber; and (iii) identify the SNP markers and the causal gene affecting seed germination ability at LT in cucumber. This study will provide a basis for elucidating the regulatory mechanisms underlying LT-tolerance in cucumber at the germination stage, and accelerate the screening of LT-tolerant varieties.

## Materials and methods

### Plant materials

A set of 151 accessions from different geographical origins (**Table S1**), was used for the GWAS analysis, and was provided by the cucumber research group at the Institute of Vegetables and Flowers, at the Chinese Academy of Agricultural Sciences in China. The 151 diverse plants genotypes were grown, each line includes 5 individuals and self-pollinated under natural field conditions in the Institute of Vegetables and Flowers, Chinese Academy of Agricultural Science at Beijing in 2018 (39°90N, 116°30E; average day/night temperatures 26/14°C; day length ~14 h in May and June). The individuals of each line were collected, and mixed seeds were further used for phenotype identification.

### Low temperature germination ability evaluation

Accessions of the 151 cucumber core germplasms were phenotyped in two different environments: a phytotron in Beijing in summer of 2019 (2019S) and an incubator in Shandong (36°51N, 118°50E) in Shandong province in summer of 2020 (2020S) (**Table S1**). Fifteen seeds per triplicate were placed at 13°C for 14 days in the dark and at 28 °C for 7 days as control (Kłosińska et al., 2013). For each experiment, three replicates were applied. Each replicate was randomly placed and the core germplasm collection was randomly placed in each replicate (block). Fifteen seeds per line were placed in a 90-mm-diameter Petri dish containing two layers of filter paper soaked 6 h with 30 ml 55°C water, then were exposed to 6 ml of distilled water. Seed germination (radicle ≥ 2 mm long) was recorded daily during the treatment stage (14 days for low temperature and 7 days for control) and radical length were measured at 14 day for low temperature and 7 day for control by Image J (Song et al., 2018). The relative value of four evaluated indices, including relative germination rate (RGR), relative germination energy (RGE), relative germination index (RGI) and relative radical length (RRL) were calculated between 13°C and 28°C (Song et al., 2018). The four indices, i.e., RGR, RGE, RGI and RRL were used for GWAS analysis.

### Statistical analysis

Statistical analysis of the phenotypic data was implemented using SAS v.9.4 (SAS Institute Inc., Cary, NC, USA, 2012). Pearson’s liner correlation coefficients were found among the four indices, and were analyzed using R software (R Core Team, 2019). An analysis of variance (ANOVA), principal component analysis (PCA) analysis was also performed in SAS v.9.4 (SAS Institute Inc., Cary, NC, USA, 2012). Membership function and comprehensive evaluation values (D-value) were analyzed in Excel 2016 (Xie, 1983; He, 2006). Principal component W_j_ = I_j_/∑I_j_ (j = 1, 2, …, n), where I_j_ represented the contribution rate of the jth principal component; Membership function value (X_j_) = (X_j_ - X_min_)/(X_max_-X_min_) (j = 1, 2, …, n), where X_j_ represented the jth principal component value, X_min_ and X_max_ represented the minimum and maximum value of the jth principal component in different germplasms, respectively; Comprehensive evaluation values D = ∑ (U_j_ × W_j_) (j = 1, 2, …, n). The D-value as a comprehensive evaluation index was used to represent the LT resistance of various germplasm. The D-value was used as an input for cluster and GWAS analysis. The sum of squares of dispersion (Ward) method was used for cluster analysis and the Euclidean distance method was used to measure in R software (R Core Team, 2019) based on the average D-value of each core germplasms in two experiments.

### Genome-wide association analysis and linkage disequilibrium analysis

The re-sequenced genome data and SNPs of the 151 accessions of the core germplasm collection are available in the NCBI Database (PRJNA831637). A FaST-LMM model (Jiang et al., 2021) was used for association tests of LT tolerance with an estimated relatedness matrix as covariate. All of the 151 genotypes were included in the GWAS. *P*=1.0 × 10^−6^ was used as the significant threshold. The linkage disequilibrium (LD) decay coefficients (*r^2^*) between high-density SNPs was calculated using Plink software (Purcell et al., 2007), and was used to evaluate LD decay (Liu et al., 2020b). Association mapping of LTG traits using SNP genotyping, population structure, relative kinship and LD analysis were identified (unpublished). Manhattan plots and the LD heatmaps were drawn by qqman package (Turner, 2014) in the R environment.

### Identification of QTLs and candidate gene analysis

The candidate locus was identified by repeatedly detected loci of various indices in two environments. The regions with significant trait-associated SNPs were described as candidate regions by unifying a set of various indices. The annotations of candidate genes in QTL regions were obtained based on “cucumber genome V3 (‘9930’ as reference genome) (http://cucurbitgenomics.org/organism/20)” (Li et al., 2019). The candidate genes were identified by the following steps: first, many genes related to abiotic stresses were predicted using Swiss-Prot (https://www.uniprot.org/)、TAIR (https://www.arabidopsis.org/index.jsp) and the gene ontology (GO) database (http://amigo.geneontology.org/amigo/landing). Secondly, SNPs in the exons or promoters of different genes, that differentiated the 47 LT-sensitive lines from the 21 LT-resistant lines were identified based on sequence alignment using MEGA X (Kumar et al., 2018). Finally, the expression level of these candidate gene was analyzed at varying time points, between germplasm differing in their LTG response, to further identify candidate genes.

### Promoter *cis*-elements analysis *and* prediction of the CsPPR protein–protein interaction network

Promoter (the 2.0 kb upstream of ATG) were used for searching regulatory elements of the *CsPPR* gene using PlantCARE online (http://bioinformatics.psb.ugent.be/webtools/plantcare/html/) (Lescot et al., 2002). And the result was visualized by TBtools (Chen et al., 2020). Cucumber orthologs of the Arabidopsis PPR proteins were identified using the Cucurbit (http://cucurbitgenomics.org/feature/gene/CsaV3_1G044080) and Arabidopsis databases (https://www.arabidopsis.org/servlets/TairObject?type=locus&name=AT4G04370). The functional interaction networks of proteins were identified by the online STRING database (https://cn.string-db.org/cgi/network?taskId=baqwil7ulR5D&sessionId=bZBjkvzPG61G).

### RNA extraction and qRT-PCR verification

A time-course expression analysis of the four sensitive lines (‘R13’, ‘R42’, ‘R77’, ‘R137’), together with the four resistant lines (‘R99’, ‘R152’, ‘R167’, ‘R174’) were examined during seed germination. Cucumber seeds exposed to 13°C in an incubator for 0 d,12 h, 1 d, 3 d and 7 days were collected. There were three biological replicates for each treatment. For *Arabidopsis thaliana*, Columbia-0 ecotype (wild-type) Col-0 and five T_1_ transgenic lines were grown 1/2 MS with hygromycin agar medium. Ten-days-old seedlings were collected for RNA extraction.

Total RNA was extracted, and the first-strand complementary DNA (cDNA) was synthesized using RNeasy Plant Mini Kit (Qiagen, Hilden, Germany) and a PrimeScript RT Reagent Kit with gDNA Eraser (TaKaRa, Kyoto, Japan), respectively, according to the manufacturer’s instructions. Quantitative reverse transcription PCR analysis (qRT-PCR) was performed using SYBR Premix Ex TaqTM II (TaKaRa, Kyoto, Japan). The PCR primers (**Table S2**) were designed with DNAMAN7.0 (Woffelman, 2004). *Actin1* (*Csa3G806800*) was employed as the reference gene to normalize gene expression values (Xie et al., 2018). Each qRT-PCR experiment was performed with three biological replicates. The analysis of gene relative expression data was performed using the 2^−ΔΔCt^ method (Livak and Schmittgen, 2001). Significant differences between different materials were detected using two-tailed, two-sample Student’s *t*-test (*p*<0.05 or *p*<0.01) in Excel (2016).

### Analysis of transgenic *A.thaliana* plants under low temperature stress

Seeds of *A.thaliana* Col-0 ecotype was obtained from the Arabidopsis Biological Resource Center (https://abrc.osu.edu/). To obtain transgenic lines in which *CsPPR* was driven by the 35S promoter, the coding sequence of *CsPPR* was amplified from cucumber and subsequently cloned to the *Bam*HI and *Pm*I I sites of the *pCAMBIA1305.4* vector to generate the *35S::CsPPR* recombinant plasmid. *Agrobacterium tumefaciens* strain GV3101 carrying *35S::CsPPR* plasmid was used to transform *A.thaliana* ecotype Columbia (Col-0) plants using the floral dip method (Clough and Bent, 1998). Transgenic seedlings were selected for their resistance to 40 mg/mL hygromycin. The resistant T_1_ transgenic seedlings were selected and propagated and T_2_ homozygotes selected for further analysis. The expression of *CsPPR* were compared between the transgenic plants with Col-0 using qRT-PCR.

Seeds of the Col-0 and transgenic plants were harvested at the same time from plants grown under the same conditions. For LT treatment, 50~100 seeds of each genotype were planted on the same 1/2MS medium and exposed to 4°C for 40 days under a 16 h-light/8-h dark cycle. At 4°C for 20 days, the number of seedlings that germinated was recorded, and the germination rate calculated. An obvious emergence of the radicle through the seed coat was used to indicate seed germination. At 4°C for 40 days, the number of surviving individuals was recorded, and the survival rate calculated. For the non-chilling control, treatment and analyses was identical to that described for the chilling treatment, except that the seeds were stored at 22°C for 5 days.

## Results

### Diversity of the RGR, RGE, RGI and RRL traits among the core cucumber germplasm

**Figure 1:**
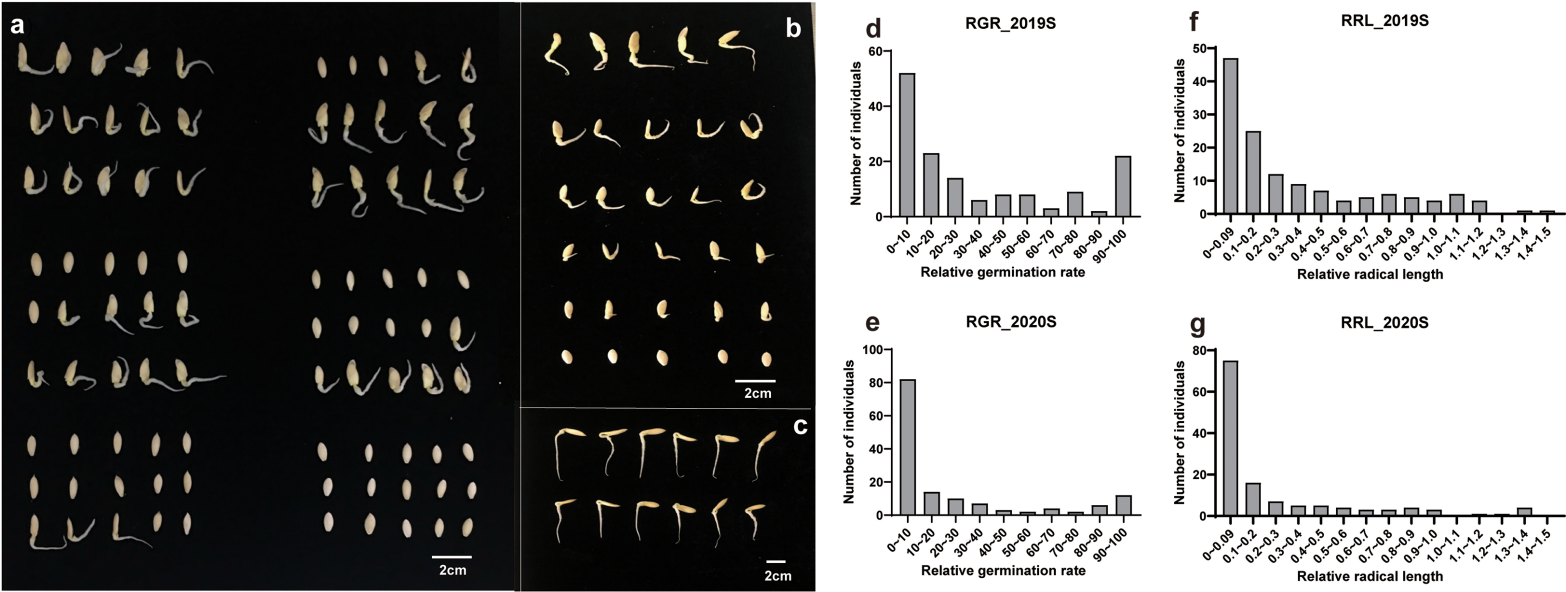
Differences in germination rate at low temperature stress among accessions from the core germplasm. **(A)** Germination rate and **(B)** radical lengths of accessions from the core germplasms incubated at 13°C. **(C)** Germination under control, i.e., 28°C, temperatures. Bars = 2 cm. **(D, E)** Relative germination rate and **(F, G)** relative radical length in 2019S and 2020S.

**Figure 2:**
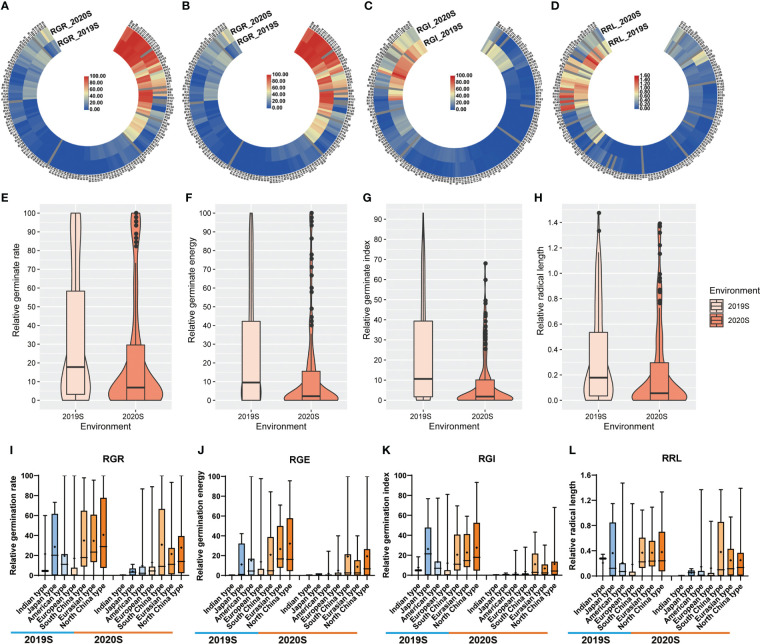
Heatmap, violin and box plots showing the phenotypic distribution of RGR, RGE, RGI and RRL among cucumber accessions grown under two environments. **(A–D)** Heatmap depicting the repeatability of the four germination indices, i.e., RGR, RGE, RGI and RRL. Blue and red bars represent accessions sensitive and tolerant to LTs, respectively, with the color intensity indicating the level of sensitivity or tolerance. **(E–H)** Violin and box plots depicting the distribution of the germination indices in two experiments. **(I–L)** Box plots depicting the distribution of the germination indices in seven ecotypes. Key: RGR, relative germination rate; RGE, relative germination energy; RGI, relative germination energy; RRL, relative radical length.

Phenotypic data related to the germination of 151 cucumber accessions of the core germplasm collection were obtained from two environments daily. From these data, four indices, i.e., RGR, RGE, RGI and RRL, were calculated (**Table S3**). Seedlings were tested in phytotrons in the summer of 2019 and in 2020, i.e., 2019S and 2020S, in Beijing and Shandong province respectively. The phenotypic data showed diverse variation among germplasms (**Figure 1**). RGR, RGE, RGI and RRL exhibited values of 0-100%, 0-96.59%, 0-75.20% and 0-1.37%, with average values of 29.01%, 21.07%, 16.24%, 0.35%, respectively (**Figures 2A–D**; **Table 1**). There was a clear bimodal distribution of RGR, RGE, RGI and RRL among the accessions, indicating that these traits were quantitative in nature, and were controlled by multiple genes (**Figures 2E–H**). In total, seven cucumber ecotypes were evaluated, including the Indian (n = 3 lines), Japanese (n = 6 lines), American (n = 13 lines), European (n = 17 lines), South China (n = 21 lines), Eurasian (n = 21 lines), and the North China ecotypes (n = 70 lines) (**Table S3**). Three market-types have higher LT resistant in the two environments, e.g., the North China, the Eurasian, and the South China types (**Figures 2I–L**). The coefficients of variation (*CV*s) of RGR, RGE, RGI and RRL were 112.05, 130.03, 119.89, and 117.12%, respectively (**Table 1**). Significant (*P* < 0.001) positive correlations were observed between each pair of the four traits, i.e., RGR, RGE, RGI, and RRL, when tested in both environments, with the correlation coefficients ranging from 0.56 (RGE_2020S and RRL_2019S) to 0.97 (RGR_2019S and RGE_2020S) (**Figure S1**). RGR, RGE, RGI in 2019S and 2020S showed high positive correlations, ranging from 0.62-0.97 (**Figure S1**). These findings indicate that the four traits probably exert a synergistic effect on cucumber seed germination at LT. Meanwhile, data collected from each of the two years were highly correlated and consistent, providing a solid foundation for GWAS analysis.

**Table 1:** Phenotype statistics of low temperature germinability traits in core germplasm.

Trait	Max	Min	Mean	SD	Skewness	Kurtosis	*CV (%)*
RGR (%)	100	0	29	32.5	1.1	-0.2	112.1
RGE (%)	96.6	0	21.1	27.4	1.3	0.4	130
RGI (%)	75.2	0	16.2	19.5	1.3	0.7	119.9
RRL (%)	1.4	0	0.3	0.3	1.2	0.3	117.1

RGR, relative germination rate; RGE, relative germination energy;

RGI, relative germination energy; RRL, relative radical length;

SD, standard deviation; CV, Coefficient of variation.

### D-value evaluation and clustering analysis

**Figure 3:**
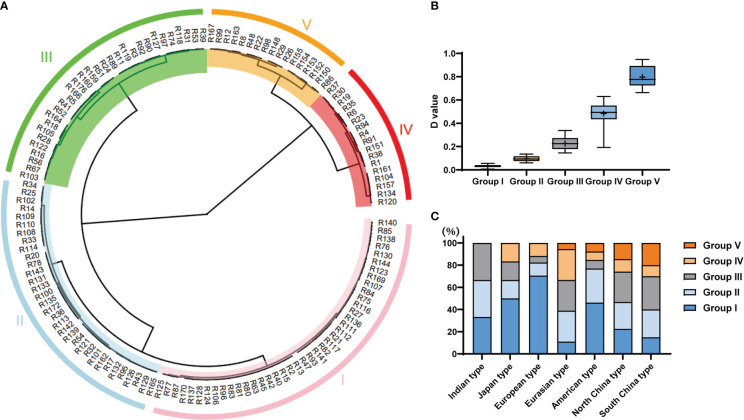
Analysis of the cucumber accessions based on their tolerance to germination at low temperatures. **(A)** Clustering of accessions of the core germplasm collection using the comprehensive D-value to indicate germination at low temperature. The I, II, III, IV and V indicate group I, group II, group III, group IV, and group V, respectively. **(B)** The range of D-values from Group I to Group V. **(C)** The proportion of Group I to Group V of D-values found within the different ecotypes.

To evaluate accurately the LT tolerance ability of core germplasms, four principal (prin) components were identified by principal component analysis. Prin1 and prin2 represented 97.59% in 2019S and 98.07% in 2020S of the four indices (**Table S4**). Weight of prin1 was 95.98%, while prin2 was 4.02% by membership function (**Table S4**). Finally, a comprehensive evaluation (D-value) of the germination ability of the core germplasm, that encompassed all four germination indices, was calculated. The line with bigger D-value was more resistant for LT. Based on the average D-value per line of two environments, these core germplasms were divided into 5 clusters (I, II, III, IV, V) by the Ward method, when Euclidean distance was 13.5 (**Figure 3A**). Cluster I consisted of highly sensitive accessions, those in Cluster II were sensitive, while Clusters III, IV and V consisted of accessions that were intermediate in tolerance, tolerant, and highly-tolerant respectively (**Table S5**; **Figure 3A**). Each cluster contained different ecotypes (**Figure 3C**). The higher the D-value, the higher the LT resistance of the germplasms (**Figure 3B**). Significant differences were found among various groups (**Figure 3B**). In general, most accessions in the Indian, Japanese and European groups were sensitive to LT, while those in the Chinese group showed higher tolerance (**Figure 3C**). However, the LTG of some germplasms differed greatly among experiments, e.g., the ‘R161’ line grouped in the highly resistant cluster in 2019S, but showed susceptibility to LTs in 2020S (**Figures 2A–D**). Thus, we combined all the phenotypic data from the two experiments to differentiate the susceptible from the resistant lines. Of the 151 tested accessions, we could define 47 as sensitive, i.e., belonging to the highly sensitive group: ‘R13’, ‘R42’, ‘R77’, and 21 as resistant, i.e., belonging to the highly tolerant group: ‘R99’, ‘R152’, ‘R167’.

### Genome-wide association analysis of low temperature resistance at germination

**Figure 4:**
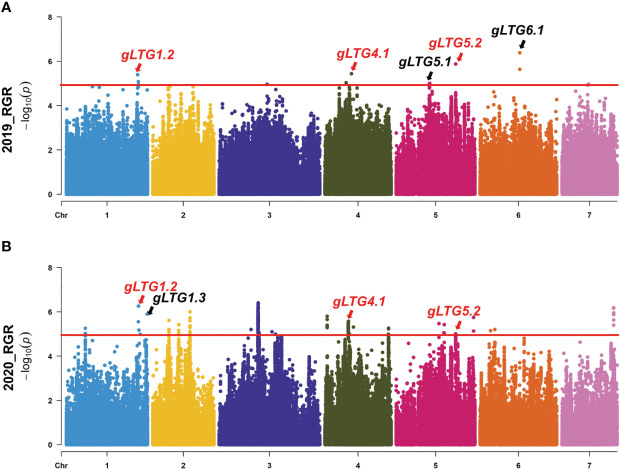
Genome-wide association analysis (GWAS) Manhattan plots of **(A)** RGR in 2019 **(B)**, and RGR in 2020. The red horizontal lines indicate the significance threshold (*P < 10^−6^*). Arrow heads indicate the position of strong peaks.

The various index (D-value, RGR, RGE, RGR and RRL) were used to repeatedly detect loci at the threshold of 5.0 across all 7 chromosomes with well-fitted quantile-quantile (Q-Q) plots in two environments. SNP loci distributed on chromosomes 1, 4, 5 and 6 were detected, and were named *gLTG1.1*, *gLTG1.2*, *gLTG1.3*, *gLTG4.1*, *gLTG5.1*, *gLTG5.2* and *gLTG6.1*, respectively (**Figure 4**, **Figure S2**, **Table 2**).

**Table 2:** The highly significant SNPs by GWAS in *gLTG1.1*, *gLTG1.2*, *gLTG1.3*, *gLTG4.1*, *gLTG5.1*, *gLTG5.2* and *gLTG6.1* in two environments.

Evaluation index	SNP	Chromosome	Position	-Log10(*P*)	Locus
2019_RGR	S1_28888267	1	28888267	5.398085219	***gLTG1.2***
	S4_10726644	4	10726644	5.438961759	***gLTG4.1***
	S5_13575438	5	13575438	5.006938639	*gLTG5.1*
	S5_24113432	5	24113432	5.878299159	***gLTG5.2***
	S6_16113098	6	16113098	6.379871503	*gLTG6.1*
2020_RGR	S1_29243248	1	29243248	6.256519753	***gLTG1.2***
	S1_32769084	1	32769084	5.905424132	*gLTG1.3*
	S4_9470362	4	9470362	5.46897565	***gLTG4.1***
	S5_24117906	5	24117906	5.008209429	***gLTG5.2***
2019_RGE	S5_13505835	5	13505835	5.47204252	*gLTG5.1*
	S5_24355383	5	24355383	8.112022481	***gLTG5.2***
2020_RGE	S1_10550485	1	10550485	5.487259593	*gLTG1.1*
	S1_29567774	1	29567774	6.897947497	***gLTG1.2***
	S1_32769084	1	32769084	7.433682161	*gLTG1.3*
	S5_24113432	5	24113432	5.26412299	***gLTG5.2***
2019_RGI	S1_10550485	1	10550485	6.454858055	*gLTG1.1*
	S5_24113432	5	24113432	5.779659366	***gLTG5.2***
	S6_16113098	6	16113098	7.14431828	*gLTG6.1*
2020_RGI	S1_10550485	1	10550485	7.370798485	*gLTG1.1*
	S1_30005894	1	30005894	6.02751893	***gLTG1.2***
	S1_32769084	1	32769084	7.653170653	*gLTG1.3*
	S4_11744432	4	11744432	5.251093917	***gLTG4.1***
	S5_13487402	5	13487402	6.307335458	*gLTG5.1*
	S5_24113432	5	24113432	5.18327183	***gLTG5.2***
	S6_16057237	6	16057237	5.215772131	*gLTG6.1*
2019_RRL	S5_24990763	5	24990763	5.46666652	***gLTG5.2***
2019_D_Value	S1_32769084	1	32769084	5.3169313	*gLTG1.3*
	S4_10306596	4	10306596	5.084358877	***gLTG4.1***
	S5_24113432	5	24113432	5.993556776	***gLTG5.2***

The italic indicated that the locus were detected more than three times by various indexes in 2019 and 2020.

**Figure 5:**
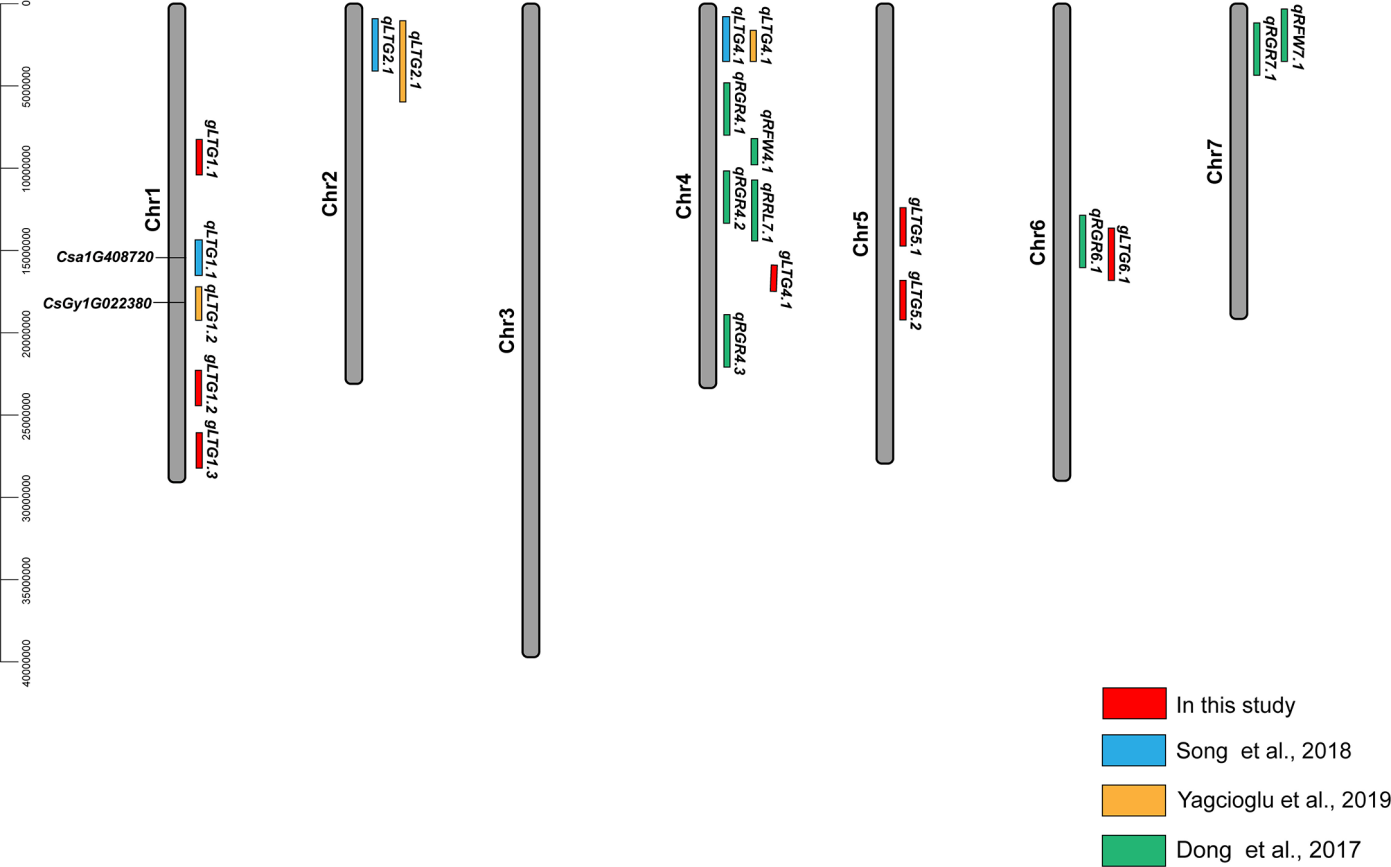
Locations of QTLs for LTG in cucumber reported in this study and previous studies (2015–2022). The red font indicates the loci of this study and their exact SNP locations can be found in (**Table S5**). The black font indicates candidate gene identified in previous studies (Li et al., 2022a).

Of these seven loci, *gLTG5.2* was detected eight times, and two loci (*gLTG1.2* and *gLTG4.1*) were detected four times, using the four indices in each of the two experiments, respectively. These loci were thus considered to have a stable effect on LTG (**Table S6**). For the rest, four loci (*gLTG1.1*, *gLTG1.3*, *gLTG5.1*, *and gLTG6.1*) were detected in three times. By comparing the SNP (single nucleotide polymorphism) positions of these 7 loci with QTLs reported in previous studies (**Table 2**), it was evident that one (*gLTG6.1*) of the 7 loci in this study colocalized with the known ones (**Figure 5**), which indicating the locus were reliable via GWAS.

### Candidate gene analysis at the novel and stable loci for LTG

The novel and stable loci on chromosomes 1 (*gLTG1.2*), 4 (*gLTG4.1*) and 5 (*gLTG5.2*) that exhibited significantly association with LT germinability (**Figure 4** **S2**; **Table S6**). So, their candidate regions were further analyzed using LD decay around the peak SNPs, haplotype analysis, functional annotation of the orthologs in *Arabidopsis* and qRT-PCR. Given that all of them were detected in the GWAS for RGR, therefore RGR is the best choice to further analyze coincidence rate between genotype and loci SNP. Based on RGR in two environments, 21 resistant lines (Group V and IV) and 47 sensitive lines (Group I and II) were used for haplotype analysis (**Table S7**; **Figure S3**). And ‘R13’, ‘R42’, ‘R77’, ‘R137’ of LT sensitive group (Group I) and ‘R99’, ‘R152’, ‘R167’, ‘R174’ of LT tolerance group (Group V) were used for expression analysis (**Figure 3A**).

**Figure 6:**
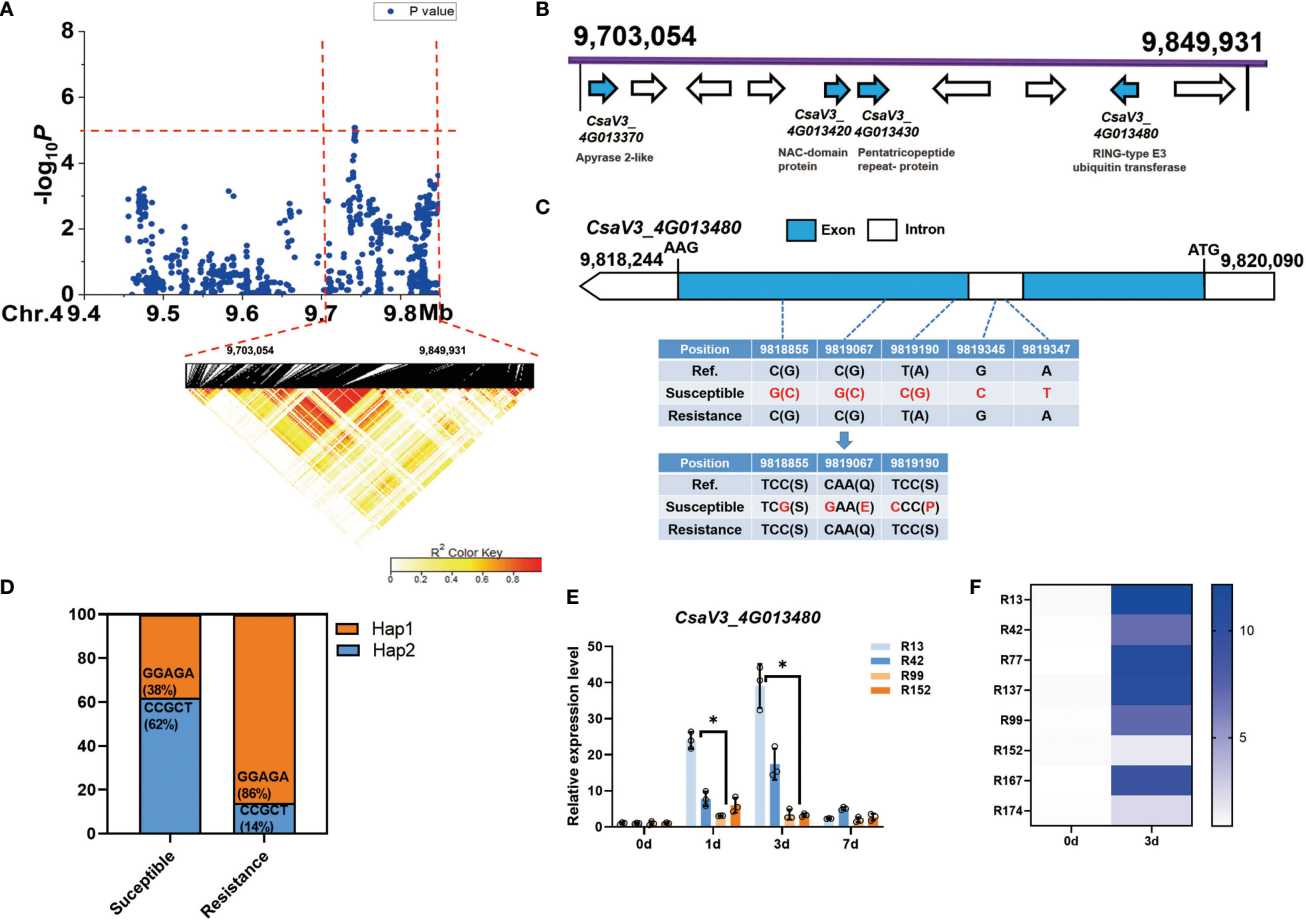
Identification of cucumber low-temperature germination trait-associated gene *CsaV3_4G013480*. **(A)** Local Manhattan plot (top) and LD heatmap (bottom) surrounding the peak on chromosome 4. The dashed line indicates the significance threshold (*P* < 10^−6^). Dashed lines indicate the candidate region (~ 146.8 kb) for the peak. **(B)** Ten functionally annotated genes were predicted in the *gLTG4.1* candidate region. The arrows represent the direction of the genes. The genes shown as blue arrows are related to abiotic stress. Gene annotation is listed below. **(C)** Gene structure of *CsaV3_4G013480*. Blue and white rectangles indicate exons and introns, respectively. **(D)** Orange represents the proportion of accessions that are Hap1 (GGAGA) while blue represents the proportion that are Hap2 (CCGCT) among the susceptible and resistant lines. **(E)** The expression level of *CsaV3_4G013480* in the LT-sensitive lines (‘R13’, ‘R42’) and LT-resistant lines (‘R99’, ‘R152’) after LT treatment (0, 1, 3 and 7 d), was tested through qRT–PCR. *Actin* was used an internal control. Data are represented as average values with the SD of three independent biological replicates. **(F)** The heatmap indicates the relative expression level of *CsaV3_4G013480* in LT-sensitive lines (‘R13’, ‘R42’, ‘R77’, ‘R137’) and LT-resistant lines (‘R99’, ‘R152’, ‘R167’, ‘R174’) after LT treatment (0 d and 3 d), tested through qRT–PCR. *Actin* was used an internal control. *Significant difference (*P <0.05*).

For the novel and stable *gLTG4.1* locus, the candidate region on Chr.4 was analyzed by pairwise LD correlations leading to a region from 9,703,054 bp to 9,849,931 bp by LD block (**Figure 6A**). Ten annotated genes are located in this region, four of which are associated with different abiotic stresses (**Figure 6B**, **Table S8**). *CsaV3_4G013370*, encodes an enzyme with ATPase and ADPase activity (an apyrase), that are involved in stress-responses in *Arabidopsis* (Kim et al., 2009); *CsaV3_4G013420*, encodes a NAC-domain containing protein, which has a role in seed germination (Dekkers et al., 2013) and plant abiotic stress responses (Nakashima et al., 2012); *CsaV3_4G013430*, encodes a pentatricopeptide repeat-containing (PPR) protein; while *CsaV3_4G013480*, encodes a RING-type E3 ubiquitin transferase, and takes part in ABA-mediated stress responses (Nakashima et al., 2012). Of these candidate genes, only *CsaV3_4G013480* was of interest since five SNPs – three in the exon and two in the intron, were found. Two of the three SNPs are predicted to lead to non-synonymous substitutions (**Figure 6C**), i.e., a Ser →Pro and a Gln→ Glu respectively (**Figure 6C**). We also found that 62% of the 47 sensitive lines carried the Hap1 “CCGCT” sequence, while 86% of the 21 resistant lines had the alternate Hap2 “GGAGA” motif (**Figure 6D**). Interestingly, *CsaV3_4G013480* expression was highly induced 1 d after LT treatment in the susceptible lines ‘R13’ and ‘R42’ while in contrast, its expression was lower in the resistant lines ‘R99’ and ‘R152’ (**Figure 6E**). Moreover, after 3 d at 13°C, *CsaV3_4G013480* expression in the susceptible lines (‘R13’ ‘R42’ ‘R77’ ‘R137’) was significantly higher than that in the resistant lines (‘R99’ ‘R152’ ‘R167’ ‘R174’) (**Figure 6F**). It is possible that haplotype and the expression of this gene might play a role in cucumber LTG response.

**Figure 7:**
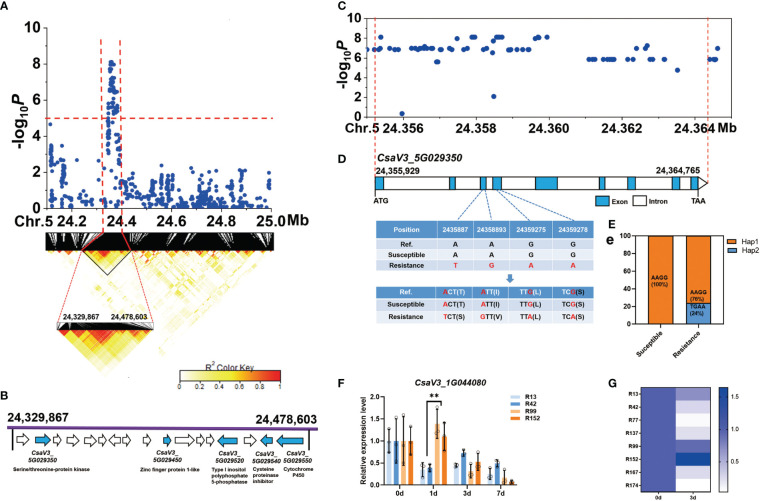
Identification of cucumber low-temperature germination trait-associated gene *CsaV3_5G029350*. **(A)** Local Manhattan plot (top) and LD heatmap (bottom) surrounding the peak on chromosome 5. The dashed line indicates the significance threshold (*P* < 10^−6^). Dashed lines indicate the candidate region (~ 148.7 kb) for the peak. **(B)** Seventeen functionally annotated genes were predicted in the *gLTG5.2* candidate region. The arrows represent the direction of the genes. The genes shown as blue arrows are related to abiotic stress and their annotation is indicated. **(C)** The corresponding DNA polymorphisms of *CsaV3_5G029350* with significant associations. **(D)** Gene structure of *CsaV3_5G029350*. Blue and white rectangles indicate exons and introns, respectively. **(E)** Bar chart showing the proportion of haplotypes - orange represents the proportion of accessions that are Hap1 (TGAA) while blue represents the proportion that are Hap2 (AAGG) among the susceptible and resistant lines, **(F)** The relative expression level of *CsaV3_5G029350* in low-temperature sensitive lines (‘R13’, ‘R42’) and low-temperature resistant materials (‘R99’, ‘R152’) after low temperature treatment (0, 1, 3 and 7 d), tested through qRT–PCR. *Actin* was used an internal control. Data are represented as average values with SD of three independent biological replicates. **(G)** The heatmap indicates relative expression level of *CsaV3_4G013480* in LT-sensitive lines (‘R13’, ‘R42’, ‘R77’, ‘R137’) and LT-resistant lines (‘R99’, ‘R152’, ‘R167’, ‘R174’) after low temperature treatment (0 and 3 d), tested through qRT–PCR. *Actin* was used an internal control. Data are represented as the average values with the SD of three independent biological replicates. **Significant difference (*P <0.01*).

For the novel and stable *gLTG5.2* locus, the common peak SNPs were further analyzed and a candidate region from 24,329,867 bp to 24,478,603 bp on Chr.5 was estimated using pairwise LD correlations (*r^2^* ≥ 0.6) (**Figure 7A**). In this region, 17 annotated genes were found, and five candidate genes related to abiotic stress identified (**Figure 7B**, **Table S8**), they include *CsaV3_5G029350* (a serine/threonine-protein kinase), *CsaV3_5G029450* (a zinc finger protein), *CsaV3_5G029520* (A type I inositol polyphosphate 5-phosphatase), *CsaV3_5G029540* (a cysteine proteinase inhibitor), *CsaV3_5G029550* (a cytochrome P450). Nevertheless, 84 SNPs significantly associated with LTG in the gene *CsaV3_5G029350* (**Figure 7D**). Two SNPs in the third exon caused a base change from A-to-T and from A-to-G, which resulted in amino acid change from Thr→Ser and Ile→Val, respectively (**Figure 7D**). Two SNPs located in the fourth exon, led to a synonymous mutation. All 47 sensitive lines were (Hap1), carried the haplotype “AAGG” while 24% of the resistant lines had the “TGAA” (Hap2) (**Figure 7E**). However, the SNP variation in the resistant lines were not consistent with the phenotype, indicating that cucumber LT germinability could be controlled by multiple genes (such as *gLTG1.2*, *gLTG4.1*, and *gLTG5.2*). The relative expression of *CsaV3_5G029350* was higher than that of the susceptible lines ‘R13’, ‘R42’ at 1 d at 13°C (**Figure 7F**). In addition, the expression level of *CsaV3_5G029350* in the susceptible lines (‘R99’ ‘R152’ ‘R167’) was significantly higher than in the susceptible resistant lines (‘R42’ ‘R77’ ‘R137’) after 3 d at 13°C (**Figure 7G**).

### GWAS revealed *CsPPR* as a regulator of LTG in cucumber

**Figure 8:**
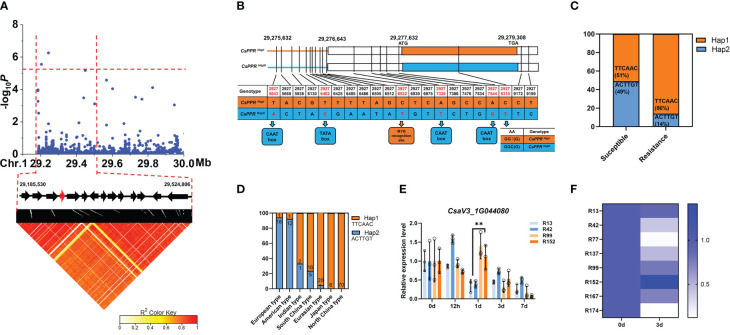
Identification of cucumber LT germination trait-associated gene *CsaV3_1G044080* (*CsPPR*). **(A)** Local Manhattan plot (top) and LD heatmap (bottom) surrounding the peak on chromosome 1. The dashed line indicates the significance threshold (-log_10_(*P*)=5.0). Dashed lines indicate the candidate region (~ 339 kb) for the peak. Fourteen functionally annotated genes were predicted in the *gLTG1.2* candidate region. The arrows represent the direction of the genes. Function of red arrow gene is relative to abiotic stress. **(B)** Schematic representation of two haplotypes of *CsPPR*. Orange and blue box represent promoter elements. **(C)** Orange represents the proportion of accessions that are Hap1 (TTCAAC) while blue represents the proportion that are Hap2 (ACTTGT) among the susceptible and resistant lines genotype. Orange represents the genotype Hap1 proportion in susceptible or resistant lines. Blue represents the genotype Hap2 proportion in susceptible or resistant lines. **(D)** The percentage of Hap1 and Hap2 genotypes within each ecotype. **(E)** Relative expression level of *CsaV3_1G044080* in LT sensitive lines (‘R13’, ‘R42’) and LT resistant lines (‘R99’, ‘R152’) after LT treatment (0 d, 12 h, 1 d, 2 d, 3 d and 7 d), tested through qRT–PCR. *Actin* was used an internal control. Data are represented as average values with SD of three independent biological replicates. **(F)** The heatmap indicates relative expression level of *CsaV3_1G044080* in LT sensitive lines (‘R13’, ‘R42’, ‘R77’, ‘R137’) and LT resistant lines (‘R99’, ‘R152’, ‘R167’, ‘R174’) after LT treatment (0 d and 3 d), tested through qRT–PCR. *Actin* was used an internal control. **Significant difference (P <0.01).

For the *gLTG1.2* locus, the signals were detected that consistently exceeded a significant threshold (−log_10_ *P* ≥ 5.0) in 2019_RGR, 2020_RGR, 2020_RGE, 2020_RGI. The haplotype blocks were estimated using Plink tool (Purcell et al., 2007) in the *gLTG1.2* locus; this effort further narrowed this region into only one haplotype block harboring significantly associated SNPs (Chr1: 29,185,530—29,524,806 bp) (**Figure 8A**). Fourteen annotated genes were located in this haplotype block (**Figure 8A**, **Table S8**). And only one gene (*CsaV3_1G044080*), encoding pentatricopeptide repeat-containing (PPR) protein (namely *CsPPR*), was reported to be related to abiotic stress. In *Arabidopsis*, *AtPPR* played a central role in abiotic stress during seed imbibition and seed development stages (Liu et al., 2016). *AtCsPPR* might interact with *AtOTP* (**Figure S2**) (Lee et al., 2009). To determine if the candidate gene *CsPPR* could function in a LTG response in cucumber, the haplotypes of 21 resistant and 47 sensitive cucumber lines were compared (**Figure S3**). Six variants of the significantly associated SNPs were identified in the 68 lines (**Figure 8B**). For 21 resistant cucumber lines and 47 sensitive cucumber accessions, 21 significantly associated SNPs located at the upstream (< 2 kb) and genic region of *CsPRR* were classified into 2 haplotypes (**Figure 8B**). Approximate 50% sensitive lines had the haplotype2 (Hap2; “ACTTGT”), while 86% resistant lines carried the Hap1 (“TTCAAC”) (**Figure 8C**). By further analysis of the accessions with different haplotypes, the “ACTTGT” (Hap2) haplotype was only existed in European type, American type, Indian type, South China type and Eurasian type (**Figure 8D**).

Of these significantly associated SNPs, in CDS region, one located at nucleotide 29,278,310 resulted in a C-to-T conversion, but led to no amino acid changes (**Figure 8B**); many *cis*-elements in *CsPPR* promoter were related to LT (**Figure S4**), eg. ABRE (ABA-responsive element), MYB (cold responsive element), ERE (ethylene-responsive element), CGTCA-motif (MeJA-responsive element), TGACG-motif (MeJA-responsive element). Of these, 5 motifs in promoter were altered by 5 SNP. SNP_29,275,643, SNP_29,277,326, SNP_29,277,544 caused change in TATA-box, SNP_29,276,402 bp caused change in TATA-box, and SNP_29276532 caused changes in MYB recognition site, respectively (**Figure 8B**). But the remaining SNPs has no change function of gene. The expression level of this gene in resistant lines (‘R99’ ‘R152’) was significantly higher than that in sensitive lines at 1 d after LT treatment (**Figure 8E**). In a larger panel of 8 randomly selected accessions, a general trend was observed that the expression level of resistant lines (‘R99’ ‘R152’ ‘R167’) were significantly higher than that of susceptible lines (‘R42’ ‘R77’ ‘R137’) at 3 d at 13°C (**Figure 8F**).

### *CsPPR* of *gLTG1.2* ectopic expression confers enhanced LT resistance during seed germination in *A. thaliana*

**Figure 9:**
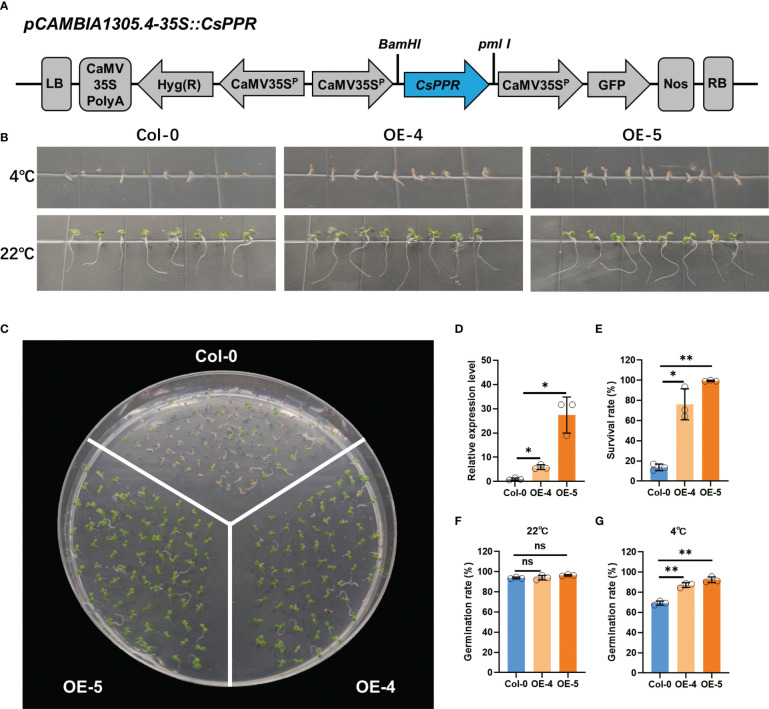
Low temperature tolerance of transgenic *A.thalina* ectopically expressing *CsPPR*. **(A)** Schematic diagram of the *CsPPR* construct for *A.thaliana* transformation. LB: Left border; CaMV35SPolyA: untranslated region of the CaMV 35S gene; *hyg* (R): Hygromycin phosphotransferase gene that confers hygromycin resistance; CaMV35Sp: CaMV 35S promoter; *CsPPR*: full-length of cucumber *CsPPR*; GFP: green fluorescent protein; NOS: transcriptional terminator sequence of the nopaline synthase gene; RB: right border. **(B)** Phenotype of the Col-0, *CsPPR*-expressing plants exposed to 4°C for 20 days and 22°C for 5 days. WT, wild-type **(C)** Phenotypes of acclimated WT, OE-4 and OE-5 plants exposed to 4°C for 40 days. At least three plates were used per treatment. **(D)** qRT-PCR showing the relative expression level of *CsPPR* in three independent *CsPPR-*ectopically expressing transgenic lines OE-4 and OE-5. Error bars represent the standard deviation of three replicates. **(E)** Survival rates after 4°C for 40 days. The data are the mean ± SD values of three independent experiments. Germination rates of the Col-0, OE-4 and OE-5 seeds germinated on 1/2 MS medium after **(F)** 5 days at 22°C, and **(G)** 20 days at 4°C. For **(F, G)** the data shown is the mean ± SD (n=50~100) of three replicates, with the error bars representing the standard deviation. The asterisks indicate the statistically significant differences of expression between control and transgenic lines. “**” represents “*p<0.01*”, “*” represents “*p<0.05*”.

To further determine the reliability of the GWAS result, we determined if candidate gene *CsPPR*, identified at the *gLTG1.2* locus, could have a role in seed germination under LT stress. A recombinant plasmid *35S::CsPPR* was introduced into *A. thaliana* by *A. tumefaciens*-mediated floral dip method (**Figure 9A**). Five homozygous T_2_ transgenic lines (OE1-5) were obtained. The transgenic lines (OE-4, OE-5) were randomly selected for further experiments.

The expression level of wild-type (Col-0), the lines ectopically expressing *CsPPR*, i.e., OE-4 and OE-5, were detected by qRT-PCR. OE-4 and OE-5 had 6-fold and 27-fold higher *PPR* transcripts compared to those in Col-0 (**Figure 9D**). Between 50-100 seeds of Col-0, OE-4 and OE-5 were germinated at 22°C for 5 days or 4°C for 40 days. Col-0 generally showed germination inhibition, and the germination rate was significantly lower than that of the *CsPPR* transgenic seeds (OE-4 and OE-5) at 4°C for 20 days (**Figures 9B, G**). In contrast, the germination rate of Col-0, OE-4 and OE-5 were same when exposed to a non-chilling temperature, i.e., 22°C for 5 days (**Figures 9B, F**). Further, the survival rates of the transgenic lines (OE-4 and OE-5) were higher than that of the Col-0 genotypes at 4°C for 40 days (**Figures 9C, E**). Interestingly, *CsPPR* expression showed a dosage effect for survival rate. For example, the expression of *CsPPR* on OE-5 had 5-fold higher transcripts than OE-4; Meanwhile, the survival rate of OE-4 was higher than Col-0 by over 63%, and the survival rate of OE-5 was 86%, higher than that of Col-0. These data were consistent between cucumber and *Arabidopsis*. Viewed collectively, these results support that *CsPPR* is very likely the causal gene to regulate LTG.

## Discussion

LT stress is one of the major abiotic stresses seriously affecting cucumber growth, development, and quality. The ability of seeds to germinate at LT is also a key factor affecting cucumber production at early stage of cucumber lifecycle. Therefore, it is urgent to identify LT-tolerant accessions, and develop cucumber varieties with LT-tolerance at the germination stage. GWAS was an efficient and effective way to excavate novel LT-tolerant genes, active during germination in cucumber.

In this study, cucumber LTG assessment was analyzed by four traits: RGR, RGE, RGI and RRL. In the association analysis, the phenotypic values of each of these traits exhibited high variation, with the *CVs* ranging from 112% to 130% (**Table 1**). The results indicated that the collected accessions represented a range of genetic backgrounds, and that they showed wide variation in the ability to germinate under LT, and were thus suitable for studying the genetic basis of LTG via GWAS.

Accurate phenotypic data is critical for successful gene mapping. In this study, phenotypic data were collected from two sites (Beijing or Shandong), two environments (phytotron or incubator) and over two years (2019 or 2020), which better ensured the reliability of the data and reduces spurious environmental effects.

Many studies including this one, have reported that the cucumber LTG trait is polygenic (Gu et al., 2002; Kłosińska et al., 2013), and have identified major or minor effect QTLs (Song et al., 2018; Yagcioglu et al., 2019). In the past five years, QTLs associated with LTG (GR, GE, GI, RL and RFW) have been identified using RILs or F_2:3_ lines. In this GWAS, QTLs for LTG tolerance were found on four chromosomes (**Figure 4**, **Table 2**), suggesting a complex genetic architecture underlying LTG in cucumber (**Figure 2**, **Table 1**), consistent with a previous study (Song et al., 2018). Among the 17 QTLs detected in the previous study, two of the three signals contributed to QTL intervals identified in this present study. QTLs *qLTG1.1* and *qLTG1.2* were close to the GWAS locus *gLTG1.2*. In addition, the GWAS locus *gLTG4.1* was located between QTLs *qLTG4.1* and *qRGR4.1*. Similar loci were detected by GWAS and QTL analysis, in genotypes of different genetic background, different segregating populations and modes of mapping, suggesting that the GWAS results were reliable. Simultaneously, more associated loci broaden the potential for breeding development.

The regulatory mechanisms of LT response are complex and dynamic processes (Ding et al., 2019; Ding and Yang, 2022; Kidokoro et al., 2022), involving the perception of signals at the cytomembrane, transduction of the signal from the cytomembrane to the cell nucleus, and the activation of regulatory mechanisms in the cell nucleus. Correspondingly, multiple factors participate in the LT regulatory response, including, transcription factors PPR (Xing et al., 2018), NAC (Zhuo et al., 2018; Liu et al., 2020a), GRAS (Wang et al., 2021; Li et al., 2022a), bZIP (Li et al., 2022b) and WRKY (Liu et al., 2022b; Sun et al., 2022a; Zhang et al., 2022; Zhao et al., 2022), phytohormones ABA (Zhu, 2016; Song et al., 2022; Zhang et al., 2023), JA (Li et al., 2021; Sun et al., 2022b) and GA (Magome et al., 2004; Achard et al., 2008) and various metabolic pathways especially those producing compatible solutes. In this study, ten genes in the GWAS regions, possibly related to abiotic stress were identified (**Table S8**). Three candidate genes were predicted as potential causal genes underlying the stable and novel loci, including *CsaV3_1G044080* for *gLTG1.2*, *CsaV3_4G013480* for *gLTG4.1* and *CsaV3_5G029350 for gLTG5.2* (**Figures 6**–**8**). These candidate genes either have significantly associated nonsynonymous SNPs which resulted in amino acid changes, or motif variations in the promoter *cis*-elements important for stress response.

(a) The *A.thaliana* orthologue of *CsaV3_4G013480*, encodes a plasma-membrane-localized RING E3 ubiquitin ligase, that regulates tolerance to cold stress (Kim and Kim, 2013). For example, *Arabidopsis* RING E3 ubiquitin ligase *AtATL7* improves cold tolerance, while *AtATL80* negatively regulate the cold tolerance response (Suh and Kim, 2015).
(b) For *gLTG5.2*, the *A. thaliana s* orthologue of *CsaV3_5G029350* encodes a serine/threonine-protein kinase. Ectopic expression of a SNF1- serine/threonine-protein kinase *TaSnRK2.4* in *A.thaliana* improved tolerance to freezing stress compared to the non-transformed, wild-type plants (Mao et al., 2010). Based on these collective data, we speculate that these three genes, i.e., *CsaV3_1G044080, CsaV3_4G013480 and CsaV3_5G029350* might be part of the cucumber seed response to LT at germination. However, the specific role, and mode of action of the products of these genes in a signal transduction response for regulating LTG requires further study.
(c) The *CsaV3_1G044080* gene, encoding a PPR family protein, is homologous to the *A.thaliana* gene *At4G04370*. In rice, the PPR protein *OsV4* plays an important role in tolerance to cold stress (Gong et al., 2014; Tan et al., 2014). *Arabidopsis* transgenic lines, overexpressing a gene for a PPR protein called *SOAR1*, were able to tolerate various stresses, including cold stress (Jiang et al., 2015). *SOAR1* positively regulated plant response to cold stress through the *CBF* signaling pathway, and ABA-dependent, and -independent signaling pathways (Jiang et al., 2015). MYB, an essential cold regulatory transcriptional factor, has a recognition *cis*-acting element at 29,277,326 bp in the promoter of *CsaV3_1G044080* (An et al., 2018). There are also additional stress-responsive *cis*-acting elements (e.g. TGACG motif) in *CsaV3_1G044080* that could be activated in response to LT and which are important in to cold resistance (Baker et al., 1994).

The sequence variation in the promoter and transcribed region of the gene(s) identified at a QTL, are crucial for linking DNA polymorphisms to a phenotypic effect. In this study, the *CsPPR* association study identified sequence variation that were significantly correlated with variation in RGR, RGE and RGI among the population studied. Of the 21 SNPs identified, SNP_29277326, within a MYB recognition site in the *CsPPR* promoter, might be the most significant. The expression levels could be affected by the genetic loci variation in promoter (Yu et al., 2019; Li et al., 2020; Zhang et al., 2020). qRT-PCR and contrasting haplotypes further identified its expression level by the effect of the promoter. The findings indicated that the promoter of resistant lines (haplotype “TTCAAC”) supported significantly higher transcriptional activity than that in the sensitive lines (**Figures 8E, F**), which was consistent with previous data that the favorable haplotypes of candidate genes were closely associated with the phenotypic performances of traits. Hence, the favorable haplotype of CDS (CsPPR^’9930’^) was transformed into *A.thaliana* and the seed germination was investigated. The seed germination rate and survival rate in transgenic *A.thaliana* were significantly higher than those in the wild-type Col-0 under LT, indicating that *CsPPR* is a causal factor in regulating seed germination in transgenic *A.thaliana* under LT. The molecular mechanism of G protein α subunit (*CsGPA1*) and heat-shock transcription factor (*CsHSFA1d*) were studied in cucumber (Qi et al., 2022; Yan et al., 2022). Thus, to verify whether, and how *CsPPR* controls cucumber seed germination and response to LT, the generation of overexpression or knockout in cucumber should be given priority.

In our study, we identified novel genes related to LTG and demonstrated that *CsPPR* contributes to cucumber LTG ability. Our results will help to facilitate future studies on the mechanism underlying LT tolerance in germinating cucumber. Meanwhile, the newly-discovered molecular markers and LT-tolerant germplasm will aid future marker-assisted breeding of cucumber.

## Data availability statement

The datasets presented in this study can be found in online repositories. The names of the repository/repositories and accession number(s) can be found in the article/**Supplementary Material**.

## Author contributions

CL drafted the manuscript. CL and SD conducted the experiments and analyzed the data. DB helped analyzed the data and drafted the manuscript. XL helped collected the data, HM helped analyzed the data. SZ and XG designed the experiments. All authors contributed to the article and approved the submitted version.
