# Supplementary material for: GWAS reveals novel loci and identifies a pentatricopeptide repeat-containing protein (CsPPR) that improves low temperature germination in cucumber
Source: Front Plant Sci. 2023 Apr 26;14:1116214. doi: 10.3389/fpls.2023.1116214 (PMC10208356; doi:10.3389/fpls.2023.1116214)
Supplement: Supplementary file 1 [file Presentation_1.pdf]

Supplementary Material

GWAS reveals novel loci and identifies a *Pentatricopeptide Repeat-Containing Protein* (*CsPPR*) that improves low temperature germination in cucumber

Caixia Li[1†], Shaoyun Dong[1†], Diane M Beckles[2], Xiaoping Liu[1], Jiantao Guan[1], Xingfang Gu[1], Han Miao[1*] , Shengping Zhang[1*]

[1] *State Key Laboratory of Vegetable Biobreeding, Institute of Vegetables and Flowers, Chinese Academy of Agricultural Sciences, Beijing, 100081, China*

[2] *Department of Plant Sciences, University of California, Davis, One Shield Avenue, Davis, CA 95616, USA;*

[#] These authors contributed equally to this work.

* Correspondence authors: zhangshengping@caas.cn; guxingfang@caas.cn

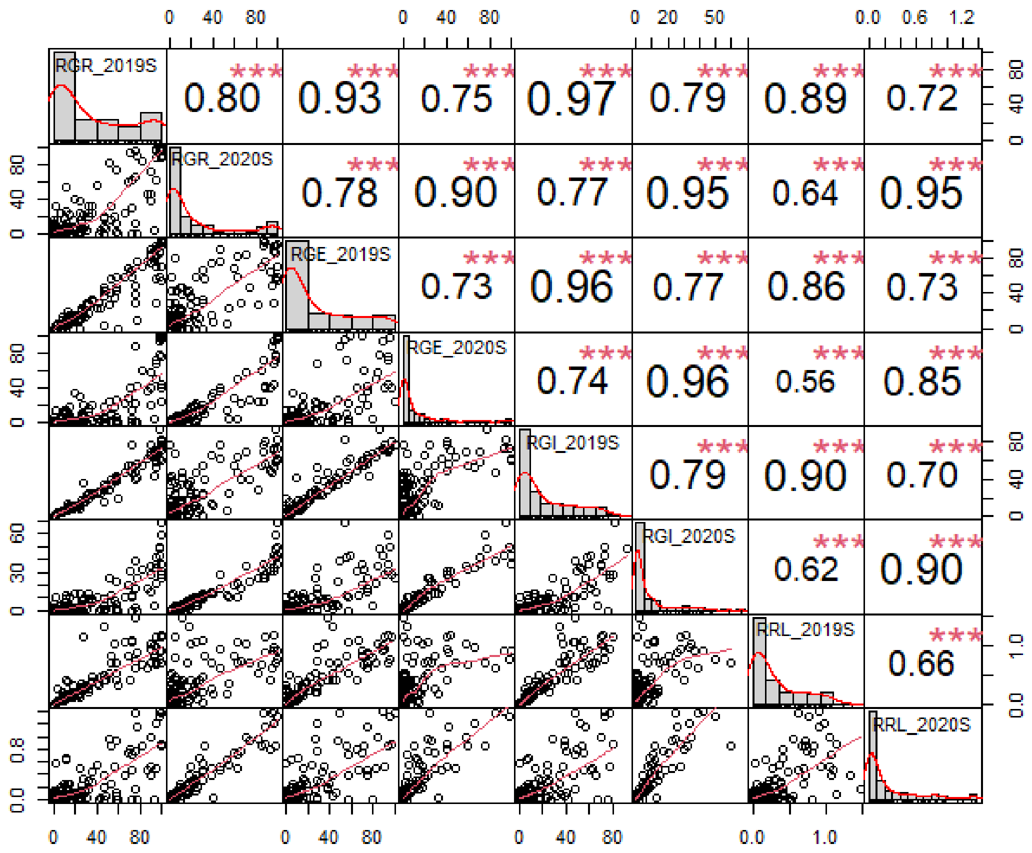

Fig. S1: Frequency distribution and spearman rank correlations of four indexes (RGR, RGE, RGI and RRL) under two environments. RGR, relative germination rate; RGE, relative germination energy; RGI, relative germination energy; RRL, relative radical length. *** indicate significance at *P<0.001*, respectively.

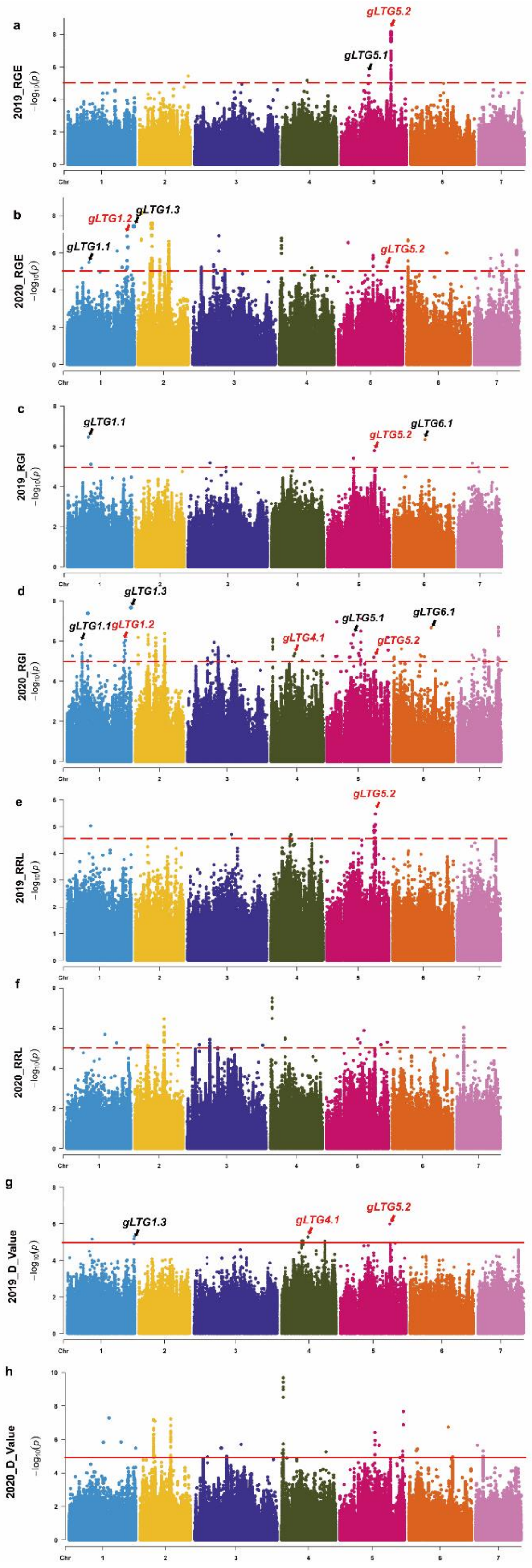

Fig. S2: Manhattan plots of GWAS for RGE (a-b), RGI (c-d), RRL (e-f) and D Value (g-h). The red dashed horizontal line indicates the significance threshold ($P < 10^{-6}$).

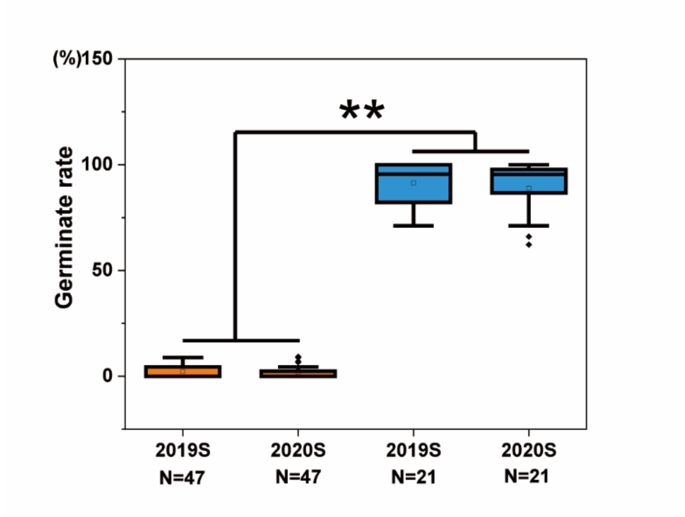

Fig.S3: The phenotype collected in two experiments.

In the box plots, the center line denotes the median, box limits are the upper and lower quartiles, and whiskers mark the range of the data. "n" indicates the number of core germplasms with the same phenotype. The significance of difference is analyzed with a two-tailed t-test.

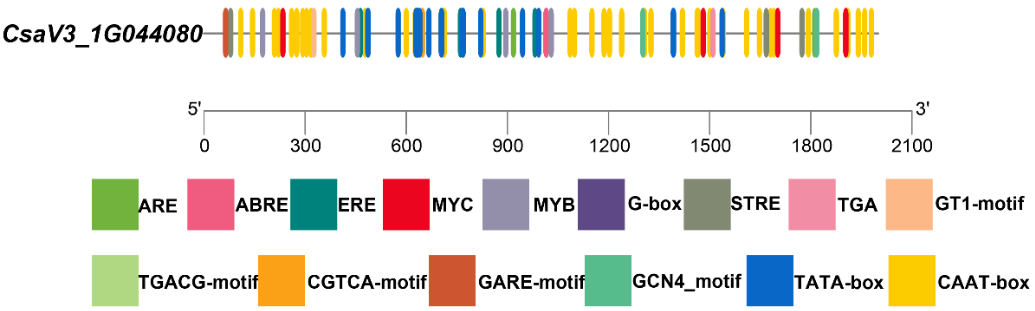

Fig.S4: The *cis*-elements of the promoter were predicted.

The bar is listed in below. Various color rectangles represent different motif.

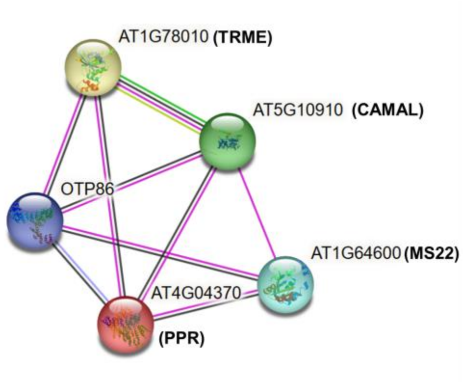

Fig.S5: Predicted protein-protein interaction network of *CsPPR* protein in cucumber based on interolog from *Arabidopsis.*

Table S1: Experimental environment of tested germplasms.
Table S2: The primers were used in this study.
Table S3: Germplasm information used in this study.
Table S4: Values of germination indicators, principal components, subordinate function, and comprehensive evaluation (D value) for germplasms.
Table S5: Difference of low temperature tolerance in germination stage for accessions in five groups.
Table S6: Summary of the significant SNPs by GWAS in *gLTG1.2*, *gLTG4.1* and *gLTG5.2* in two environments.
Table S7: The selected highly resistant and susceptible lines.
Table S8: Candidate genes in region of *gLTG1.2*, *gLTG4.1* and *gLTG5.2*.
Table S9: The SNP haplotypes of the selected highly resistant and susceptible lines in *gLTG1.2*, *gLTG4.2* and *gLTG5.2*.
